# Erythromycin Modification That Improves Its Acidic Stability while Optimizing It for Local Drug Delivery

**DOI:** 10.3390/antibiotics6020011

**Published:** 2017-04-25

**Authors:** Erika L. Cyphert, Jaqueline D. Wallat, Jonathan K. Pokorski, Horst A. von Recum

**Affiliations:** 1 Department of Biomedical Engineering, Case Western Reserve University, 10900 Euclid Avenue, Cleveland, OH 44106, USA; elc50@case.edu; 2 Department of Macromolecular Science and Engineering, Case Western Reserve University, 2100 Adelbert Road, Cleveland, OH 44106, USA; jdw114@case.edu (J.D.W.); jon.pokorski@case.edu (J.K.P.)

**Keywords:** erythromycin, infection, pH-sensitive, pH-responsive, hydrophobic, adamantane, cyclodextrin, polymer

## Abstract

The antibiotic erythromycin has limited efficacy and bioavailability due to its instability and conversion under acidic conditions via an intramolecular dehydration reaction. To improve the stability of erythromycin, several analogs have been developed—such as azithromycin and clarithromycin—which decrease the rate of intramolecular dehydration. We set out to build upon this prior work by developing a conjugate of erythromycin with improved pH stability, bioavailability, and preferential release from a drug delivery system directly at the low pH of an infection site. To develop this new drug conjugate, adamantane-1-carbohydrazide was covalently attached to erythromycin via a pH-degradable hydrazone bond. Since *Staphylococcus aureus* infection sites are slightly acidic, the hydrazone bond will undergo hydrolysis liberating erythromycin directly at the infection site. The adamantane group provides interaction with the drug delivery system. This local delivery strategy has the potential of reducing off-target and systemic side-effects. This work demonstrates the synthesis of a pH-cleavable, erythromycin conjugate that retains the inherent antimicrobial activity of erythromycin, has an increased hydrophobicity, and improved stability in acidic conditions; thereby enhancing erythromycin’s bioavailability while simultaneously reducing its toxicity.

## 1. Introduction

Erythromycin (EM) is a macrolide antibiotic that is frequently used in the treatment of *Staphylococcus aureus* (*S. aureus*) infections and is a common alternative for patients with penicillin allergies [1,2]. Generally, EM has a low cytotoxicity; however, it has been reported to cause gastrointestinal problems as well as liver toxicity due to its instability and chemical conversion under acidic conditions [1,3]. To improve the acid-stability of macrolide antibiotics, different analogs have been developed such as azithromycin and clarithromycin [1]. While azithromycin and clarithromycin have an improved acidic stability compared to EM, all three macrolides nevertheless undergo an acid-mediated chemical conversion via the slow loss of cladinose sugar from the 14-membered aglycone ring [4,5]. We therefore set out to develop a modified, more acid-stable form of EM to minimize these toxicities as well as increase its hydrophobicity slightly to improve EM’s overall bioavailability at an infection site, and which can be combined with a smart drug delivery system.

In an effort to improve the bioavailability and pharmacokinetic properties of antibiotics, a variety of chemical conjugations have been employed. Specifically, ciprofloxacin and vancomycin have been chemically modified with hydrolytic ((acyloxy)alkyl ester) and different aliphatic and aromatic linkers (cathelicidin-related antimicrobial peptides) in order to enable a targeted antibiotic delivery and to enhance the antibacterial activity (i.e., improved ability to penetrate the bacterial membrane) of the original drug [6,7]. Furthermore, several different analogs of clarithromycin and β-lactam antibiotics have been synthesized that are less susceptible towards developing resistance to certain bacterial strains [8,9]. Amino acid sequences have also been utilized to chemically modify antibiotics in order to increase the drug’s hydrophobicity and concentration at the target delivery site [10].

Adamantane (AD) is a hydrophobic molecule that has frequently been used to chemically modify drugs to improve a variety of drug properties [11]. Specifically, AD has been conjugated to a variety of central nervous system drugs and antiviral agents in an effort to improve their hydrophobicity, drug stability, pharmacokinetics, and clinical efficacy [11]. In another application, AD has been conjugated to chemotherapeutic agents to maximize their therapeutic efficacy while minimizing their hepatic and cardiotoxicities [12,13,14]. Non-steroidal anti-inflammatory drugs (NSAIDs) and diacylglycerol acyltransferase 1 inhibitors (DGAT1) have also utilized AD modifications to increase the potency of NSAIDs and to create a novel DGAT1 inhibitor drug for more effective diabetic treatment [15,16]. Generally, when AD is conjugated to drugs, it functions to increase the drug’s hydrophobicity which can enable the drug to bind to tissue more readily. With more drug binding to tissue, AD thereby increases the tissue residence time of the drug and its therapeutic effects [17,18]. Similar strategy can be used to retain the AD conjugated drugs in a drug delivery system.

Several methods have been used to improve the acidic stability and therapeutic efficacy of EM. When EM is in an acidic environment it degrades via an intramolecular dehydration reaction and forms the compound anhydroerythromycin A which is an inactive and more toxic form of the drug [19]. Several analogs of EM (azithromycin and clarithromycin) have been developed that have a chemical substitution at the location where internal dehydration is first initiated to prevent the degradation reaction from starting, resulting in slightly increased acidic stability and therapeutic efficacy [19,20,21]. However, these analogs are still susceptible to acid degradation. Several techniques have been used to improve the acid stability and bioavailability of the analogs. For clarithromycin, amorphous solid dispersions have been created using cellulose acetate adipate propionate that has a high affinity for hydrophobic drugs in order to enhance the clarithromycin’s bioavailability [22]. Furthermore, alkalizers including MgO and Na_2_CO_3_ have been used to manipulate the microenvironment of clarithromycin to improve its stability and solubility when combined in a crystalline-solid dispersion system using polyvinylpyrrolidone and hydroxypropylmethylcellulose [23]. For EM, enteric coatings have been developed to create delayed-release tablets that ‘shield’ EM from acidic degradation while in the stomach [24]. Furthermore, pH-responsive polymers have also been used to improve the acidic stability of EM [25]. While these methods improve the efficacy and acidic stability of EM, none of the current studies have developed a form of EM capable of demonstrating increased acidic stability while simultaneously optimizing the drug to remain at the infection site, such as through drug delivery.

To create a more acid-stable form of EM, we chemically linked adamantane-1-carbohydrazide (AD) to EM via hydrazone chemistry [13] to create the modified drug AD-EM. Because *S. aureus* infection sites are slightly acidic [25] they would be able to cleave the acid-sensitive hydrazone bond and liberate unmodified EM. When administered locally, this would increase the residence time at the infection site, reduce availability to healthy tissues, and decrease EM’s toxicity. Our further interest is in stimulus-responsive local delivery, so the AD-EM was tested to bind to our high-affinity cyclodextrin-based polymer delivery system at physiologically normal pH and to release free drug during infection in a pH-dependent manner. 

To further elaborate, our primary motivation for developing a form of EM with a degradable linkage was to create a more stable form of EM that is able to return to its original active form when EM is liberated upon cleavage of the linkage. Further, we wanted to improve the tissue residence time of EM locally at the infection site by increasing its hydrophobicity and its affinity for our drug delivery system. With an increased hydrophobicity, the modified EM is able to remain in our drug delivery system and therefore in the tissue for a longer period of time (compared to normal EM), allowing the degradable linkage to be cleaved under the slightly acidic infection conditions and liberating the normal EM locally to the infection site.

In this paper, we have developed a synthesis procedure for adamantane-modified erythromycin (AD-EM), characterized AD-EM with Fourier transform infrared spectroscopy (FTIR), Nuclear Magnetic Spectroscopy (^1^H NMR), and Matrix Assisted Laser Desorption Ionization (MALDI) mass spectrometry, determined the hydrophobicity and solubility of AD-EM in several solvents, and evaluated the stability of AD-EM in acidic conditions. We used a zone of inhibition assay against *S. aureus* to evaluate the antibacterial activity of AD-EM compared to EM and completed bacterial biofilm penetration/biofilm quantification studies using both drugs. We also completed a preliminary drug release study from our cyclodextrin-polymer drug delivery system using both AD-EM and EM in neutral and slightly acidic conditions.

## 2. Results and Discussion

### 2.1. Synthesis of Adamantane-Modified EM (AD-EM)

**Figure 1:**
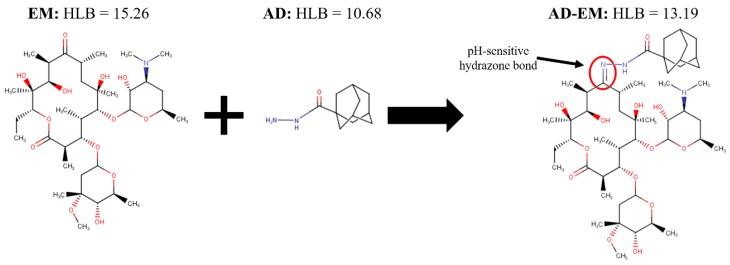
Schematic of the chemical structure and synthesis of adamantane-modified erythromycin (AD-EM). In the synthesis of AD-EM, adamantane (AD) is chemically linked to EM via a pH-sensitive hydrazone bond (C = N) at the site of the C-9 primary ketone on EM.

By chemically linking adamantane (AD) to the C-9 primary ketone of EM, a hydrazone bond is formed that is cleavable in slightly acidic environments (Figure 1) [13]. The chemical modification is specifically designed to improve the stability of EM in acidic conditions, since the AD is linked at the site where the intramolecular dehydration chemical conversion reaction of EM originates [19]. Because the hydrazone bond is cleaved in acidic conditions, the modified drug is optimized to preferentially respond to the acidic environments of infection. This is clinically relevant because *S. aureus* infections demonstrate a slightly lower pH compared to normal physiological conditions [26]. The hypothesis is that AD-EM will be capable of maximizing the antibiotic concentration of locally administered EM at the infection site, thereby reducing associated systemic toxicities and improving the therapeutic efficacy of EM. The AD moiety has previously been shown to have a high affinity (binding constant) and stably bind to cyclodextrin-based polymer systems [14,27], such as our drug delivery platform. The goal in designing the chemistry of AD-EM was to optimize the drug such that it had an improved stability while demonstrating a high affinity for cyclodextrin to facilitate a controlled pH-responsive release of EM directly at the infection site.

### 2.2. FTIR Spectrum of AD, EM, and AD-EM

**Figure 2:**
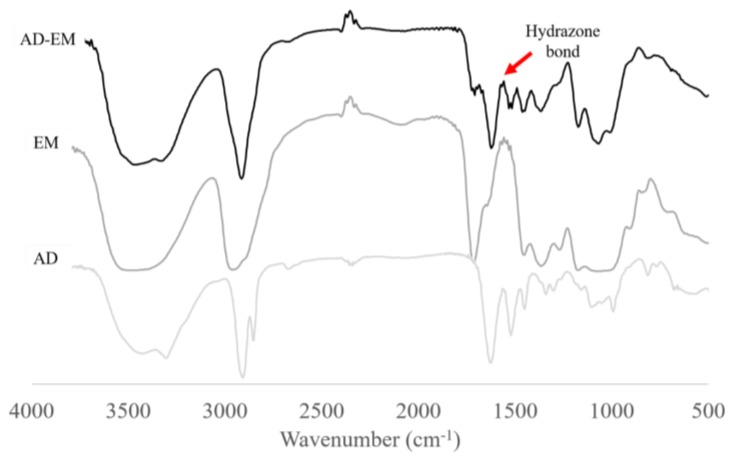
Fourier transform infrared spectroscopy (FTIR) spectra of AD-EM, EM, and AD superimposed. The chemical modification of EM to AD-EM was validated based upon the appearance of the pH-sensitive hydrazone bond peak at 1560–1570 cm^−1^ on the AD-EM FTIR spectrum that is not present in either of the spectra of AD or EM.

FTIR spectra were generated for AD, EM, and AD-EM from 500–4000 cm^−1^. The spectra were superimposed upon one another in order to evaluate whether the hydrazone bond was successfully created between AD and EM. The formation of the hydrazone bond is indicated by a peak over the span of 1560–1570 cm^−1^ on the AD-EM spectrum that is not present on the other spectra [14,28]. Figure 2 depicts the appearance of the hydrazone peak (1560–1570 cm^−1^) on the spectrum of AD-EM validating the formation and presence of the hydrazone bond.

### 2.3. ^1^H NMR of EM and AD-EM

^1^H NMR was used to confirm the presence of AD in the AD-EM (Figure S1). Both EM and AD-EM were dissolved in deuterated dimethyl sulfoxide (DMSO-d_6_) and spectra acquired. The spectra are in agreement with the reported literature spectra [29], thus we looked to see changes in the spectra following the conjugation reaction. The processed spectra of each species were compared to monitor structural differences between protons from EM and AD-EM. The AD-EM spectra confirms the presence of both AD and EM, as well as hydrazone formation, indicating successful reaction. In the NMR spectra of AD-EM, AD attachment is confirmed by the presence of two multiplet peaks at approximately 1.9 ppm and from 1.6–1.8 ppm, corresponding to protons from the fused cyclohexane rings of AD. In the spectra of the AD-EM, a singlet peak, corresponding to a hydrazone, appears at 9.0 ppm. These protons are attributed to AD-EM conjugate, since AD alone is completely insoluble in DMSO. Of note, AD-EM remained slightly insoluble in DMSO-d_6_, resulting in minor peak shape distortions, poorer resolution of peak splitting and peak broadening, for resonance peaks corresponding to protons from EM. However, peak placement is consistent between AD-EM and EM, indicating the presence of both species in the AD-EM conjugate.

### 2.4. MALDI Mass Spectrometry of EM and AD-EM

MALDI mass spectrometry was used to elucidate the molecular mass of AD-EM. The MALDI spectra of AD-EM was compared to EM. From the MALDI spectra of AD-EM, two chemical species are present. One species corresponds to the mass of the conjugate (AD-EM) (910 m/z), while the other corresponds to the mass of the EM (733 m/z) (Figure S2). It is possible that the labile hydrazone bond fragments during ionization, hence the biomodal distribution [30,31]. To further probe if ionization was responsible for the two chemical species, thin layer chromatography of AD-EM was carried out and showed evidence of two species indicating incomplete conjugation (data not shown). Although hydrazones are relatively stable, they can undergo spontaneous hydrolytic cleavage which would afford free EM and AD [32]. Alternatively, it is possible that inefficient purification resulted in residual unreacted EM in the conjugate mixture.

### 2.5. Hydrophilic-Lipophilic Balance Calculations

The hydrophilic-lipophilic balance (HLB) numbers were calculated for AD, EM, and conjugated AD-EM using three different methods (ChemAxon, Davies, and Griffin) in ChemAxon MarvinSketch Version 16.10.31 software with the HLB Predictor plug-in (See Table S1). The reported HLB numbers for each molecule were calculated using the ChemAxon method, which is an optimized weighted combination of both the Davies and Griffin methods [33]. A larger HLB number indicates a more hydrophilic molecule. The conjugated AD-EM molecule was more hydrophobic than the unmodified EM (AD-EM = 13.19; EM = 15.26) due to the addition of the hydrophobic AD group (AD = 10.68). Similar patterns were observed when the Davies and Griffin methods were used to calculate the HLB numbers where the AD-EM molecule was more hydrophobic than unmodified EM (See Table S1). When the HLB values obtained through the software are compared to experimental results, they have been shown to correlate with an R^2^ value of 0.79 [33]. Therefore, we believe that the HLB numbers that we obtained through this modeling software are a good representation of the anticipated experimental values of the HLB numbers.

### 2.6. Solubility Study

The solubility of AD-EM compared to EM was evaluated in several different aqueous buffers (i.e., PBS, Water, Acetate pH 5.0) and polar solvents (i.e., methanol, ethyl acetate, acetone). The solubility study presented in Table 1 further verifies that modified AD-EM is more hydrophobic than EM due to the addition of the hydrophobic AD group. AD-EM demonstrated a decrease in solubility in aqueous solutions compared to EM (PBS: AD-EM = 0.34 mg/mL, EM = 1.6 mg/mL; Water: AD-EM = 0.33 mg/mL, EM = 1.5 mg/mL), a decreased solubility in methanol, but showed comparable solubility to EM in several polar solvents (~40 mg/mL). This indicates that the increased hydrophobicity from the modification had a relatively large impact on its aqueous solubility, but the increase in hydrophobicity was not large enough to dramatically impact its solubility in some polar protic and aprotic solvents. The increased hydrophobicity of AD-EM is desirable to enhance its ability to bind to tissue, thereby increasing its tissue residence time and therapeutic activity.

### 2.7. Acidic Stability Spectral Scan Analysis

**Figure 3:**
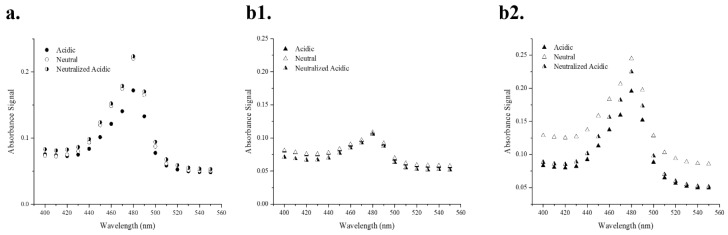
(**a**) Absorbance spectral scan of EM in acidic and neutral environments (t = several hours). (**b**) Absorbance spectral scan of AD-EM in acidic and neutral environments (t = several hours (**b1**); t = several days (**b2**)). In acidic conditions, EM is converted to an inactive form that demonstrates a lower absorbance signal than the original EM. When it is later placed in neutral conditions, the original absorbance signal in neutral conditions is restored.

The stability of EM and AD-EM in slightly acidic conditions (pH 5.0) was analyzed by studying the absorbance signal of the drug solution over a range of wavelengths (Figure 3). This technique capitalizes on the differences in absorbance signal between active EM and the major inactive (but reversible) acid-conversion product of EM—anhydroerythromycin A—which has a lower absorbance signal than active EM [4,34]. Figure 3a validates the hypothesis that EM in an acidic solution has a lower absorbance signal (0.17) compared to the drug in a neutral solution (0.22). Further, it shows that when the acidic solution of EM is later neutralized, the absorbance signal is restored back to the absorbance value in neutral conditions (0.22), presumably due to the reversible nature of the anhydroerythromycin A conversion. This restoration of signal intensity further validates the predicted model where inactive anhydroerythromycin A is in equilibrium with active EM and can be converted back to active EM in a neutralized solution [4]. A noticeable acidic conversion of EM (Figure 3a) was observed over a period of several hours.

The acidic stability of AD-EM was evaluated in Figure 3b1,b2. In Figure 3b1, the experiment was conducted over a period of several hours (the same amount of time in which a noticeable acidic conversion of EM was observed). There were no distinguishable differences observed in the absorbance patterns between the three solutions (AD-EM in acidic, neutral, and acidic later neutralized conditions) over the same period of time in which acidic conversion occurred in EM. The lack of acidic conversion in AD-EM over the same period of time as EM conversion demonstrates that AD-EM has an increased stability compared to EM in slightly acidic environments. We hypothesize that the increased acidic stability of AD-EM is due to the addition of the chemical modification at the location where the intramolecular dehydration reaction initially occurs in EM [4,34].

The same experiment as Figure 3b1 was also conducted over a longer period of time (i.e., several days instead of hours) (Figure 3b2). It was observed that, after several days, AD-EM in acidic conditions has a lower absorbance signal than in neutral conditions, demonstrating eventual loss of the adamantane and conversion of AD-EM into anhydroerythromycin A which has a lower absorbance signal in acidic conditions [34]. When AD-EM was first placed in an acidic solution and later neutralized, it showed an increased absorbance signal compared to the signal of AD-EM in acidic conditions, but was not equivalent to the original signal in neutral conditions. As stated above, this is most likely due to the reversible conversion through anhydroerythromycin A and back again. The signal of AD-EM under these conditions (i.e., initially acid and later neutralized), however, was not fully returned to the original signal of AD-EM in neutral conditions. We hypothesize that this is due to the irreversible transformation that AD-EM encounters under these conditions. Specifically, the acid cleavable linker (hydrazone bond) that was chemically added to EM to create AD-EM is irreversibly broken in acidic conditions to liberate active EM. Under neutral conditions this linker is not cleaved and the absorbance signal is higher. However, once this hydrazone bond is cleaved in acidic conditions, EM is liberated and the absorbance signal decreases (due to the intramolecular dehydration of EM). The initial absorbance signal in neutral conditions cannot be fully restored, even if EM is returned to its active form, since the bond between AD and EM cannot be re-formed.

The underlying hypothesis is that the acid-mediated conversion of AD-EM occurs over a longer period of time compared to EM, due to the addition of the linker at the location where intramolecular dehydration first occurs. While AD-EM ultimately is converted into anhydroerythromycin A in acidic conditions, the addition of the adamantane group delays the onset of acid-mediated conversion of EM. The improved acidic stability of AD-EM can help to reduce some of the toxicities and gastrointestinal side-effects that are commonly associated with the instability of EM in acidic conditions and enhance the bioavailability of active drug at a local injection site.

Colorimetric absorbance spectroscopy was determined to be the optimal and efficient method to track the acid-stability of EM and AD-EM. Once EM is reacted with concentrated sulfuric acid (27 N), the sugar groups are hydrolyzed and a yellow chromophore results that can be detected with absorbance spectroscopy [35,36,37].

### 2.8. Zone of Inhibition Antibacterial Study

**Figure 4:**
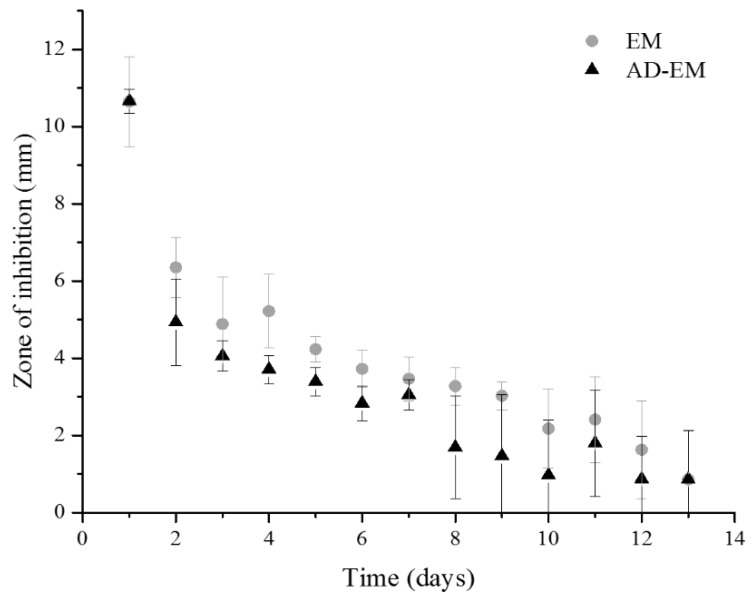
Zone of inhibition study of AD-EM and EM against *S. aureus*. AD-EM and EM demonstrated comparable activity against the growth of *S. aureus* for nearly 2 weeks. Therefore, the chemical modification did not alter the intrinsic antibacterial activity of EM.

A zone of inhibition study against *S. aureus* was conducted for approximately two weeks using EM and AD-EM loaded into insoluble cyclodextrin polymer disks to evaluate whether the chemical modification altered the intrinsic antimicrobial activity of EM (Figure 4). Both AD-EM and EM demonstrated comparable activity to inhibit the growth of *S. aureus* during the duration of the study. It is possible that the slight decrease in antibacterial activity after 8 days in AD-EM (compared to EM) can be attributed to the presence of both species in the final AD-EM composition. However, despite the presence of both species in AD-EM, we concluded that the chemical modification to EM did not significantly affect the antibacterial activity of EM.

### 2.9. Bacterial Biofilm Penetration Studies

Through the bacterial (*S. aureus*) biofilm penetration studies using both the modified (AD-EM) and EM, we observed that both the modified and unmodified drug were capable of achieving a comparable penetration through the bacterial biofilm. The ability to penetrate through the biofilm is related to the number of bacteria remaining in the biofilm after exposure to a drug compared to the control condition (90% PBS/10% DMSO). The control condition was set-up in such a way that the biofilm coated cyclodextrin polymer was placed in a solution under the same conditions used to make the drug-containing solutions. The colonies remaining in the biofilms after 1, 7, and 24 h after being treated with AD-EM, EM, or the control solution are reported in Table 2, where the remaining colonies from drug-treated biofilms are compared to the number of colonies in a control solution. After only 1 h of exposure to the drug (AD-EM or EM), nearly 30% of the original colonies in the biofilm were eradicated, and after 7 h of drug (AD-EM or EM) exposure >85% of the original colonies were killed. Interestingly, after 24 h of exposure to the drug only a small amount of additional killing occurred (~2%) compared to the 7 h time point. We hypothesize that this could be due to presence of planktonic bacteria naturally resistant to antibiotic effect, as well as the development of drug-resistant bacteria that are unable to be fully eradicated even after extended periods of exposure time to the drug. This latter hypothesis was further validated when we carried out the same experiment to 48 and 72 h using non-modified EM and approximately the same number of colonies remained even after 72 h of exposure to EM (data not shown). Given the relative comparable activity of each drug (AD-EM and EM) against *S. aureus* in the mature biofilms combined with the zone of inhibition data, we further confirm that the chemical modification to EM did not alter the intrinsic antibacterial activity of EM and that both drugs (AD-EM and EM) are capable of penetrating and killing the majority of the bacteria in a mature biofilm within 7 h. High variability in the studies reflects both the inconsistent nature of the biofilm as well as difficulty recovering bacteria to make accurate counts.

### 2.10. pH-Dependent Drug Release Study

**Figure 5:**
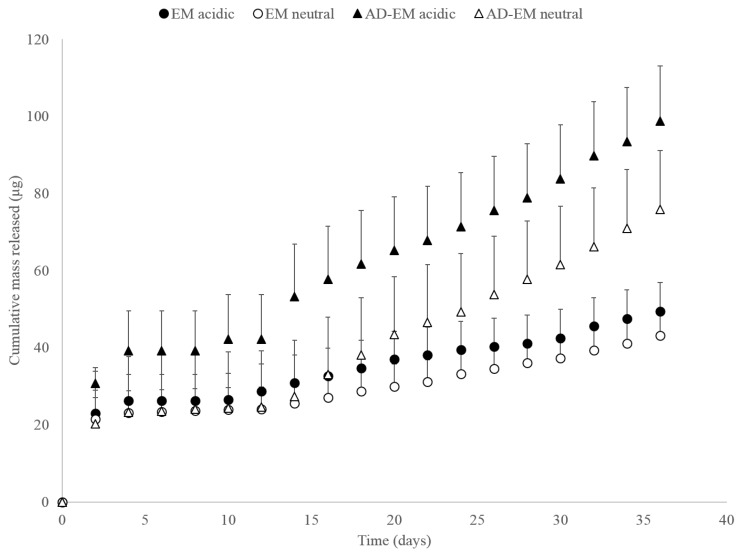
Cumulative mass of AD-EM and EM released in both acidic (pH 5.0) and neutral (pH 7.4) conditions.

As a proof-of-concept to evaluate the acid-labile modification, AD-EM and unmodified EM were loaded into insoluble cyclodextrin polymer disks, and drug release studies were conducted in both neutral and slightly acidic conditions to simulate both normal physiological conditions and the microenvironment of an infection. The slightly acidic conditions were necessary to provide the pH-stimulus required to cleave the hydrazone bond of AD-EM. Over a period of approximately 40 days, AD-EM loaded disks demonstrated a slightly increased release profile in acidic conditions compared to neutral conditions, which is attributed to the addition of the pH-sensitive linker (Figure 5). Conversely, the EM loaded polymer disks demonstrated comparable and consistent release profiles in both acidic and neutral conditions. Since EM lacked the pH-sensitive hydrazone bond, the release profiles were similar regardless of the pH condition, unlike release from AD-EM loaded disks. As we had anticipated for a pH-responsive drug delivery system, we observed an accelerated release rate of AD-EM in acidic conditions and a much slower release in neutral (presumably non-infection) conditions. This further maximizes (in addition to improved drug stability and tissue residence) the therapeutic delivery of EM at the acidic infection site when compared to delivery to pH neutral non-infected tissues.

While the difference in release rate between the AD-EM in acidic and neutral conditions may not be large, there is a more noticeable difference in the AD-EM release rates compared to the EM in both pH conditions. We hypothesize that the limited difference in release rate between AD-EM in neutral and acidic conditions is due to the relatively high binding energy of even the unmodified erythromycin for beta-cyclodextrin (−9.0 kcal/mol) [38]. Due to the high binding energy between the drug and polymer without the additional high-affinity AD moiety, it is hypothesized that with the addition of this group there is no longer a 1:1 binding interaction between the drug and polymer, but potentially a 2:1 binding interaction with the higher binding energy of that complex. Therefore, due to this complex interaction, a large amount of drug may still be trapped in the matrix of the polymer even after an extended period of time and the release results shown in Figure 5 may only demonstrate a release of the drug from the surface of the polymer. Additionally, it is possible that the mixed (2 species) composition of AD-EM also contributed to the small difference in acidic release conditions. Yet, even with the mixed composition of AD-EM, we were still able to observe slight differences in release in acidic conditions. Furthermore, due to an uncertain amount of drug still remaining in the polymer after the nearly 40 day release study, it is challenging to determine the total amount of drug initially loaded into the polymer necessary to construct a plot of total percent of drug released from the polymer.

Overall, our proof-of-concept release study demonstrates a small difference in these release rates as initially hypothesized. However, future release studies are planned that will work to optimize the release conditions and that take the large binding energy into account with the goal of demonstrating a more statistically significant difference in the release rate of AD-EM in acidic and neutral conditions.

Through Figure 5, we were also able to conclude that we were able to achieve higher levels of drug loading into the polymers with AD-EM compared to EM. This observation was consistent between the drugs released in each pH condition. We hypothesize that, with AD-EM, we can drive higher levels of drug loading into the insoluble cyclodextrin polymers due to the extra high-affinity AD group that unmodified EM lacks. To treat long-term infections clinically, it may be desirable to load a large amount of drug into the polymers to prolong the drug release time and minimize the need for additional antibiotic doses. Therefore, since AD-EM is capable of achieving increased loading over EM into our polymers it is a promising candidate for long-term antibacterial therapeutic applications.

## 3. Materials and Methods

### 3.1. Synthesis of Adamantane-Modified EM (AD-EM)

The process for synthesizing AD-EM was based upon a published protocol previously used to synthesize adamantane-modified doxorubicin but slightly modified for working with EM [13]. Specifically, 67 mg of adamantane-1-carbohydrazide (Matrix Scientific, Columbia, SC, USA) and 100 mg of erythromycin (Fisher Scientific, Pittsburgh, PA, USA) were dissolved in 50 mL methanol. Afterwards, 50 μL of trifluoroacetic acid (TFA) was used to catalyze the reaction and the solution was refluxed in an oil bath at 50 °C for 48 h in the darkness. The methanol was evaporated off using rotary evaporation and the precipitate was covered in foil and allowed to cool overnight. A mixture of 60:40% hexane:ethyl acetate solvent was used to precipitate out the unreacted AD. The solution was poured through a vacuum filter to remove the unreacted AD. The solvent mixture was evaporated off of the dissolved AD-EM with a rotary evaporator. The final AD-EM was washed with 100% hexane and vacuum filtered to remove the TFA. The purified drug was dried under vacuum and transferred to storage in a cool and dark environment.

### 3.2. FTIR Study

Small samples (<1 mg) of dried AD-EM, EM, and AD were each ground into a fine powder with a mortar and pestle. Dried potassium bromide (KBr, 0.1 g) was added to the powdered drug sample and ground into a fine powder. Each sample was placed in a 13 mm die and pressed with a force of 10 metric tons for 10 min. Each pellet was removed from the die and scanned in an Excaliber Series-BioRad FTS3000MX spectrometer. Then, 200 background and 400 sample scans were collected from 500–4000 cm^−1^. Varian Resolution Pro Software was used to transform the data using ratio and transmittance transformations. A boxcar function was used to smooth out each plot.

### 3.3. ^1^H NMR of EM and AD-EM

EM (approximately 40 mg) and AD-EM (approximately 40 mg) were dissolved in 500 µL of DMSO-d_6_, and sonication was used to promote dissolution. Samples were analyzed on a 600 MHz Varian Nuclear Magnetic Resonance Spectrometer, using 128 scans and a 45 degree pulse angle. Spectra were analyzed using MesReNova software using the residual solvent peak from DMSO-d_6_ as the reference.

### 3.4. MALDI of EM and AD-EM

EM (0.5 mg) and AD-EM (0.5 mg) were dissolved in 100 µL of acetonitrile. A saturated solution of alpha-cyano-4-hydroxy-cinnamic acid (CHCA) in acetonitrile was prepared. MALDI samples were prepared according to a three-layer method [39]: 1 µL of CHCA solution was cast onto the MALDI plate and allowed to dry; once dry, 1 µL of EM and AD-EM were cast atop the CHCA matrix and allowed to dry; once dry, an additional 1 µL of CHCA was added. MALDI was conducted on a Bruker Autoflex III operated in linear positive ion mode.

### 3.5. Hydrophilic-Lipophilic Balance Calculations

The hydrophilic-lipophilic balance (HLB) numbers of AD, EM, and the conjugate drug AD-EM were determined using ChemAxon MarvinSketch modeling software (Version 16.10.31, ChemAxon, 2016). The chemical structure of each molecule was drawn in the program and the HLB numbers were calculated using the HLB predictor plug-in using three different methods (ChemAxon, Davies, and Griffin; data for each method reported in Table S1). The larger the HLB number, the more hydrophilic the molecule. The ChemAxon method is an optimized weighted combination of the Davies and Griffin method [33]. In the Davies method, the computation is based upon different group numbers that are assigned to hydrophilic or lipophilic structural groups in a molecule [40,41]. The following equation is used in the calculation: HLB = 7 + Σ(hydrophilic group numbers) + Σ(lipophilic group numbers) [40,41]. With this method alone, it can be challenging to calculate the HLB if a group number is not assigned to a particular structural group [41]. In the Griffin method, the computation is based upon saponification (measure of fatty acids; S) and acid (A) indices of different structural groups; HLB $=20\left(1-\frac{S}{A}\right)$ [42,43].

### 3.6. Solubility Study

Small samples of both EM and AD-EM (1–3 mg samples for aqueous solvents (water, PBS (pH 7.4), and acetate buffer (pH 5.0)); 10 mg samples for methanol, ethyl acetate, and acetone) were placed in individual vials. Each solvent was gradually added to either the EM or AD-EM samples in 50–100 µL increments until the drug sample was completely dissolved and the solution was homogeneous [44,45]. Three trials were completed for each drug in each solvent and the results are reported as an average of these three trials. The solubility limit of each drug was determined to be the minimum amount of volume of each solvent where the drug was completely soluble.

### 3.7. Acidic Conversion Spectral Scans

Small samples of EM were dissolved in 2 separate solutions: neutral phosphate buffered saline (PBS) and acetate pH 5.0 buffer. The PBS solution was diluted to a concentration of 70 μM and concentrated sulfuric acid (27 N) was added to the solution in a volume equal to that of the total volume of the initial 70 µM solution to elicit a color change detectable with absorbance spectroscopy [34]. Two separate solutions were initially created with the acetate buffer. One solution was diluted to 70 μM and allowed to sit for several hours at room temperature. The other acetate solution was diluted to a slightly higher concentration, sat for 1 h at room temperature, was neutralized with sodium hydroxide (0.1 N) (such that the concentration of the solution was 70 µM), and sat for several more hours at room temperature. Both acetate solutions were treated with equal volumes of sulfuric acid to elicit a color change. After the addition of sulfuric acid, all solutions had a final concentration of 35 μM and were immediately scanned with absorbance spectroscopy (Biotek^TM^ 96 well plate reader, H1; Winooski, VT, USA) over the wavelength range of 300–700 nm in steps of 10 nm. Similar scans were collected for EM without sulfuric acid and of AD-EM over the same and a longer time frame with sulfuric acid (i.e., hours and days).

### 3.8. Synthesis of Insoluble Cyclodextrin Polymer Disks and Antibiotic Loading

Synthesis of insoluble cyclodextrin polymer disks was carried out according to a previously published protocol by Thatiparti et al. [46]. Briefly, 1 g cyclodextrin (CycloLab, Budapest, Hungary) was dissolved in 4 mL dimethylformamide (DMF) and stirred. The solution was cross-linked with hexamethylene diisocyanate (HDI; Sigma Aldrich, St. Louis, MO, USA) in a molar ratio of 1:0.16 (glucose residue: HDI). The solution was cured at 70 °C for 45 min and punched into 8 mm disks. Disks were washed to removed unreacted products over several days in 100% DMF, 50:50 DMF:water, and 100% water before use. Six disks were placed in a solution of 1.25 mg/mL AD-EM in DMF or a solution of EM in the same concentration. Solutions were covered in foil and placed on a laboratory shaker for 96 h. Loaded disks were removed from the solutions and air-dried at room temperature for later use.

### 3.9. Zone of Inhibition Antibacterial Study

The antibacterial activity of EM and AD-EM loaded insoluble cyclodextrin polymer disks was evaluated against *S. aureus* (GFP labeled *S. aureus* kindly provided by Ed Greenfield, Case Western Reserve University, Cleveland, OH, USA) according to the protocol outlined by Thatiparti et al. [46]. Briefly, fresh 70 μL fresh *S. aureus* culture was spread on a Trypticase soy agar plate. Each antibiotic loaded disk was placed in the center of a freshly seeded *S. aureus* plate and incubated overnight at 37 °C. After 24 h, the zone of inhibition (clearance) around each disk was measured using calipers and averaged. Each disk was transferred onto a freshly seeded *S. aureus* plate and placed in the incubator. This process was repeated for approximately 2 weeks, when the zones of inhibition were <2 mm.

### 3.10. Bacterial Biofilm Penetration Study

5 mm insoluble cyclodextrin polymer disks were placed in 3 mL solutions of 2× Trypticase soy broth with 30 μL of freshly cultured GFP-labeled *S. aureus*. Solutions were incubated at 37 °C for 72 h to form a mature biofilm. Each condition was set-up in triplicate. Following incubation, polymer disks were removed from the bacterial culture, excess broth removed, and placed in a 1.25 mg/mL drug solution of either EM or AD-EM dissolved in PBS with 10% DMSO. As a control, biofilm coated disks were also placed in a solution of just PBS and 10% DMSO. Each solution was placed in a laboratory shaker at 37 °C for either 1, 7, or 24 h. After this time, disks were removed from the drug solution, dried off, and placed in 4 mL of sterile Trypticase soy broth and homogenized for 30 s with an Omni TH homogenizer. Then, 70 μL of each solution was spread in duplicate on Trypticase soy agar plates and incubated overnight. Bacterial colonies were then counted and averaged using ImageJ software. Bacterial eradication was calculated as the percentage of colonies remaining in drug treated samples after different time points compared to the control colonies at that time point.

### 3.11. Proof-of-Concept Drug Release Study

Firstly, 5 mg samples of AD-EM and EM were each dissolved in 4 mL DMF. Six insoluble cyclodextrin disks were added to each solution and the samples were covered in foil and placed on an agitator for 96 h. Each drug loaded disk was washed with MilliQ water and dried at room temperature overnight. Dried disks were transferred into individual vials containing either 1 mL PBS (pH 7.4) or acetate (pH 5.0). All four conditions were set-up in triplicate. Every 48 h, all of the solution surrounding each loaded disk was removed and replenished with fresh buffer to maintain infinite sink conditions. The acidic release samples were neutralized with 0.1 N sodium hydroxide and allowed to sit overnight. To elicit a colorimetric change in the drug and enable it to be detected using absorbance spectroscopy, a volume of 27 N sulfuric acid (equal amount to the volume of the release sample) was added to each solution (acidic and neutral solutions). Aliquots of the release solution were scanned with absorbance spectroscopy using a Biotek^TM^ microplate reader at 480 nm. This process was continued for a period of 36 days. Standard curves were created with known amounts of EM and AD-EM under all of the test conditions (with NaOH and sulfuric acid). All of the dilutions (neutralization with NaOH and addition of sulfuric acid) were accounted for in the calculation of the concentration of the drug at each time point during the release study.

## 4. Conclusions

In this paper, we have created a chemically-modified, more acid stable, hydrophobic form of EM (AD-EM) that demonstrates comparable antibacterial activity against *S. aureus* as unmodified EM. Due to its increased hydrophobicity, we hypothesize that AD-EM has an increased resident time in both our cyclodextrin polymers and in tissues enabling us to capitalize on its degradable linkage which, when cleaved, liberates free EM locally at the acidic (i.e., infection) site. Given that the addition of the degradable linkage to EM does not affect the antibacterial activity of EM both in conjugated (AD-EM) and liberated (EM) form, our future work includes developing a modified form of EM with a non-degradable linkage. We believe a non-degradable linkage may ultimately be a more desirable form of EM to minimize the potential toxicity concerns with non-drug molecules that may result following the cleavage of the linkage and liberation of EM.

## Figures and Tables

**Table 1:** Relative solubility (n = 3) of EM and AD-EM in several different aqueous buffers and organic solvents.

Solvent	EM (mg/mL)	AD-EM (mg/mL)
Water	1.5 ± 0.2	0.33 ± 0.03
Phosphate Buffered Saline (PBS), pH 7.4	1.6 ± 0.4	0.34 ± 0.01
Acetate buffer, pH 5.0	15	1.39 ± 0.04
Methanol	>40	7.3 ± 0.2
Ethyl Acetate	>40	>40
Acetone	>40	37.5

**Table 2:** Quantification of bacterial colonies remaining in mature (72 h) *S. aureus* biofilms after exposure to AD-EM, EM, or a control solution (90% PBS/10% DMSO), calculated as a percentage of the colonies remaining from the control solution at each time point.

Drug Incubation Time (Hours)	AD-EM (% Control Colonies* Remaining)	EM (% Control Colonies* Remaining)
1	62.7% ± 22.1%	71.6% ± 40.2%
7	14.8% ± 13.7%	10.5% ± 6.8%
24	11.7% ± 7.9%	8.3% ± 6.6%

* Control colonies quantified after 1, 7, and 24 h incubation in PBS with 10% DMSO.

## Supplementary Materials

The following are available online at http://www.mdpi.com/2079-6382/6/2/11/s1, Figure S1: ^1^H NMR spectra AD-EM and EM in DMSO-d_6_, Figure S2: MALDI spectra of EM and AD-EM, Table S1: Hydrophilic-Lipophilic Balance calculations for AD, EM, and AD-EM using three different calculation methods.

## References

[1] Alvarez-Elcoro S., Enzler M.J. (1999). The macrolides: Erythromycin, clarithromycin, and azithromycin. Mayo Clin. Proc..

[2] White R.J. (1994). Why use erythromycin?. Thorax.

[3] Braun P. (1969). Hepatotoxicity of erythromycin. J. Infect. Dis..

[4] Hassanzadeh A., Barber J., Morris G.A., Gorry P.A. (2007). Mechanism for the degradation of erythromycin A and erythromycin A 2′-ethyl succinate in acidic aqueous solution. J. Phys. Chem. A.

[5] Noguchi S., Takiyama K., Fujiki S., Iwao Y., Miura K., Itai S. (2014). Polymorphic transformation of antibiotic clarithromycin under acidic condition. J. Pharm. Sci..

[6] Zheng T., Nolan E.M. (2015). Evaluation of (acyloxy)alkyl ester linkers for antibiotic release from siderophore-antibiotic conjugates. Bioorganic Med. Chem. Lett..

[7] Mishra N.M., Briers Y., Lamberigts C., Steenackers H., Robijns S., Landuyt B., Vanderleyden J., Schoofs L., Lavigne R., Luyten W. (2015). Evaluation of the antibacterial and antibiofilm activities of novel CRAMP-vancomycin conjugates with diverse linkers. Org. Biomol. Chem..

[8] Zhu D., Xu Y., Liu Y., Chen X., Zhao Z., Lei P. (2013). Synthesis of 4″-O-desosaminyl clarithromycin derivatives and their anti-bacterial activities. Bioorganic Med. Chem. Lett..

[9] Lewandowski E.M., Skiba J., Torelli N.J., Rajnisz A., Solecka J., Kowalski K., Chen Y. (2015). Antibacterial properties and atomic resolution X-ray complex crystal structure of a ruthenocene conjugated β-lactam antibiotic. Chem. Commun..

[10] Ibrahim M.A., Panda S.S., Birs A.S., Serrano J.C., Gonzalez C.F., Alamry K.A., Katritzky A.R. (2014). Synthesis and antibacterial evaluation of amino acid-antibiotic conjugates. Bioorganic Med. Chem. Lett..

[11] Wanka L., Iqbal K., Schreiner P.R. (2013). The lipophilic bullet hits the targets: Medicinal chemistry of adamantane derivatives. Chem. Rev..

[12] Zefirova O.N., Nurieva E.V., Shishov D.V., Baskin I.I., Fuchs F., Lemcke H., Schrӧder F., Weiss D.G., Zefirov N.S., Kuznetsov S.A. (2011). Synthesis and SAR requirements of adamantane-colchicine conjugates with both microtubule depolymerizing and tubulin clustering activities. Bioorganic Med. Chem..

[13] Luo G.F., Xu X.D., Zhang J., Yang J., Gong Y.H., Lei Q., Jia H.Z., Li C., Zhuo R.X., Zhang X.Z. (2012). Encapsulation of an adamantane-doxorubicin prodrug in pH-responsive polysaccharide capsules for controlled release. ACS Appl. Mater. Interfaces.

[14] Cyphert E.L., Fu A.S., von Recum H.A. (2017). Chemotherapeutic delivery using pH-responsive, affinity-based release. Exp. Biol. Med..

[15] Kouatly O., Geronikaki A., Kamoutsis C., Hadjipavlou-Litina D., Eleftheriou P. (2009). Adamantane derivatives of thiazolyl-*N*-substituted amide, as possible non-steroidal anti-inflammatory agents. Eur. J. Med. Chem..

[16] Pagire S.H., Pagire H.S., Lee G.B., Han S.J., Kwak H.J., Kim J.Y., Kim K.Y., Rhee S.D., Ryu J.I., Song J.S. (2015). Discovery and optimization of adamantane carboxylic acid derivatives as potent diacylglycerol acyltransferase 1 inhibitors for the potential treatment of obesity and diabetes. Eur. J. Med. Chem..

[17] Creel C.J., Lovich M.A., Edelman E.R. (2000). Arterial paclitaxel distribution and deposition. Circ. Res..

[18] Copeland R.A. (2016). The drug-target residence time model: A 10-year retrospective. Nat. Rev. Drug Discov..

[19] Fiese E.F., Steffen S.H. (1990). Comparison of the acid stability of azithromycin and erythromycin A. J. Antimicrob. Chemother..

[20] Hill D.R. (2007). 9-Hydrazone and 9-Azine Erythromycin Derivatives and a Process for Making the Same. U.S. Patent.

[21] Whitman M.S., Tunkel A.R. (1992). Azithromycin and clarithromycin: Overview and comparison with erythromycin. Infect. Control Hosp. Epidemiol..

[22] Pereira J.M., Mejia-Ariza R., Ilevbare G.A., McGettigan H.E., Sriranganathan N., Taylor L.S., Davis R.M., Edgar K.J. (2013). Interplay of degradation, dissolution and stabilization of clarithromycin and its amorphous solid dispersions. Mol. Pharm..

[23] Park J.-B., Park Y.-J., Kang C.-Y., Lee B.-J. (2015). Modulation of microenvironmental pH and utilization of alkalizers in crystalline solid dispersions for enhanced solubility and stability of clarithromycin. Arch. Pharm. Res..

[24] Ogwal S.T.U.X. (2001). Bioavailability and stability of erythromycin delayed release tablets. Afr. Health Sci..

[25] Zhang H., Wu H., Fan L., Li F., Gu C., Jia M. (2009). Preparation and characteristics of pH-sensitive derivated dextran hydrogel nanoparticles. Polym. Compos..

[26] Bronner S., Monteil H., Prévost G. (2004). Regulation of virulence determinants in *Staphylococcus aureus*: Complexity and applications. FEMS Microbiol. Rev..

[27] Granadero D., Bordello J., Perez-Alvite M.J., Novo M., Al-Soufi W. (2010). Host-guest complexation studied fluorescence correlation spectroscopy: Adamantane-cyclodextrin inclusion. Int. J. Mol. Sci..

[28] Belskaya N.P., Dehaen W., Bakulev V.A. (2010). Synthesis and properties of hydrazones bearing amide, thioamide, and amidine functions. Online J. Org. Chem..

[29] Everett J.R., Tyler J.W. (1985). An analysis of the ^1^H and ^13^C N.M.R. spectra of erythromycin a using two-dimensional methods. J. Chem. Soc. Perkin Trans..

[30] Crisalli P., Hernández A.R., Kool E.T. (2012). Fluorescence quenchers for hydrazone and oxime orthoganol bioconjugation. Bioconjug. Chem..

[31] Carpenter C.A., Kenar J.A., Price N. (2010). Preparation of saturated and unsaturated fatty acid hydrazides and long chain C-glycoside ketohydrazones. Green Chem..

[32] Kalia J, Raines R.T. (2008). Hydrolytic stability of hydrazones and oximes. Agnew. Chem. Int. Ed. Engl..

[33] Szisz D. (2015). HLB Predictor. ChemAxon Docs.

[34] Van den Bossche L., Lodi A., Schaar J., Shaakov S., Zorzan M., Tranquillini M.E., Overballe-Petersen C., Hoogmartens J., Adams E. (2010). An interlaboratory study on the suitability of a gradient LC-UV method as a compendial method for the determination of erythromycin and its related substances. J. Pharm. Biomed. Anal..

[35] Ford J.H., Prescott G.C., Hinman J.W., Caron E.L. (1953). Colorimetric determination of erythromycin. Anal. Chem..

[36] Danielson N.D., Holeman J.A., Bristol D.C., Kirzner D.H. (1993). Simple methods for the qualitative identification and quantitative determination of macrolide antibiotics. J. Pharm. Biomed. Anal..

[37] Gallagher P.A., Danielson N.D. (1995). Colorimetric determination of macrolide antibiotics using ferric ion. Talanta.

[38] Rivera-Delgado E., Ward E., von Recum H.A. (2016). Providing sustained transgene induction through affinity-based drug delivery. J. Biomed. Mater. Res. A.

[39] Keller B.O., Li L. (2006). Three-layer matrix/sample preparation method for MALDI MS analysis of low nanomolar protein samples. J. Am. Soc. Mass Spectrom..

[40] Davies J.T. (1957). A quantitative kinetic theory of emulsion type, I. Physical chemistry of the emulsifying agent. Proceedings of the Second International Congress of Surface Activity.

[41] Guo X., Rong Z., Ying X. (2006). Calculation of hydrophile-lipophile balance for polyethoxylated surfactants by group contribution method. J. Colloid Interface Sci..

[42] Griffin W.C. (1954). Calculation of HLB values of non-ionic surfactants. J. Soc. Cosmet. Chem..

[43] Kabri T., Arab-Tehrany E., Belhaj N., Linder M. (2011). Physico-chemical characterization of nano-emulsions in cosmetic matrix enriched on omega-3. J. Nanobiotechnol..

[44] Manna P.K., Kumaran V., Mohanta G.P., Manvalan R. (2004). Preparation and evaluation of a new erythromycin derivative-erythromycin taurate. Acta Pharm..

[45] Varanda F., Pratas de Melo M.J., Caço A.I., Dohrn R., Makrydaki F.A., Voutsas E., Tassios D., Marrucho I.M. (2006). Solubility of antibiotics in different solvents. 1. Hydrochloride forms of tetracycline, moxifloxacin, and ciprofloxacin. Ind. Eng. Chem. Res..

[46] Thatiparti T.R., von Recum H.A. (2010). Cyclodextrin complexation for affinity-based antibiotic delivery. Macromol. Biosci..

