# Supplementary material for: Erythromycin Modification That Improves Its Acidic Stability while Optimizing It for Local Drug Delivery
Source: Antibiotics (Basel). 2017 Apr 25;6(2):11. doi: 10.3390/antibiotics6020011 (PMC5485444; doi:10.3390/antibiotics6020011)
Supplement: Supplementary file 1 [file antibiotics-06-00011-s001.pdf]

# Supplementary Materials: Erythromycin Modification that Improves Its Acidic Stability While Optimizing It for Local Drug Delivery

**Erika L. Cyphert, Jacqueline D. Wallat, Jonathan K. Pokorski and Horst A. von Recum**

**Table S1.** Hydrophilic-Lipophilic Balance calculations for AD, EM, and AD-EM using three different calculation methods.

| Method | AD | EM | AD-EM |
|---|---|---|---|
| ChemAxon | 10.68 | 15.26 | 13.19 |
| Davies | 13.75 | 18.18 | 15.53 |
| Griffin | 6.08 | 10.88 | 9.68 |

Note: Values determined using MarvinSketch Version 16.10.31 software (ChemAxon, 2016), HLB Predictor plug-in.

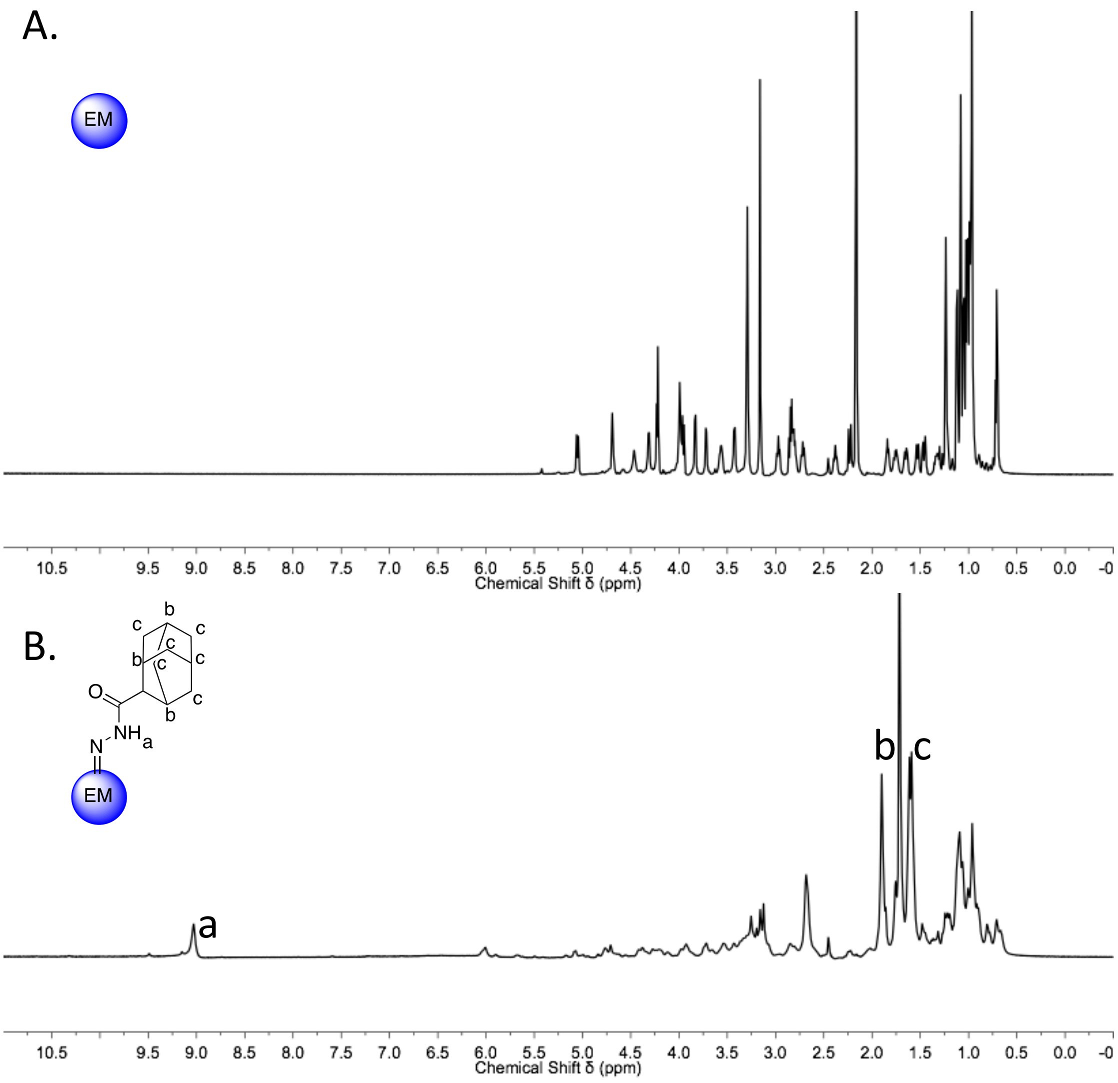


**Figure S1.** $^1$H NMR spectra (**A**). AD-EM and (**B**). EM in DMSO-d$_6$. Labels (**a**,**b**,**c**) indicate resonance peaks corresponding to AD attached to the AD-EM conjugate.

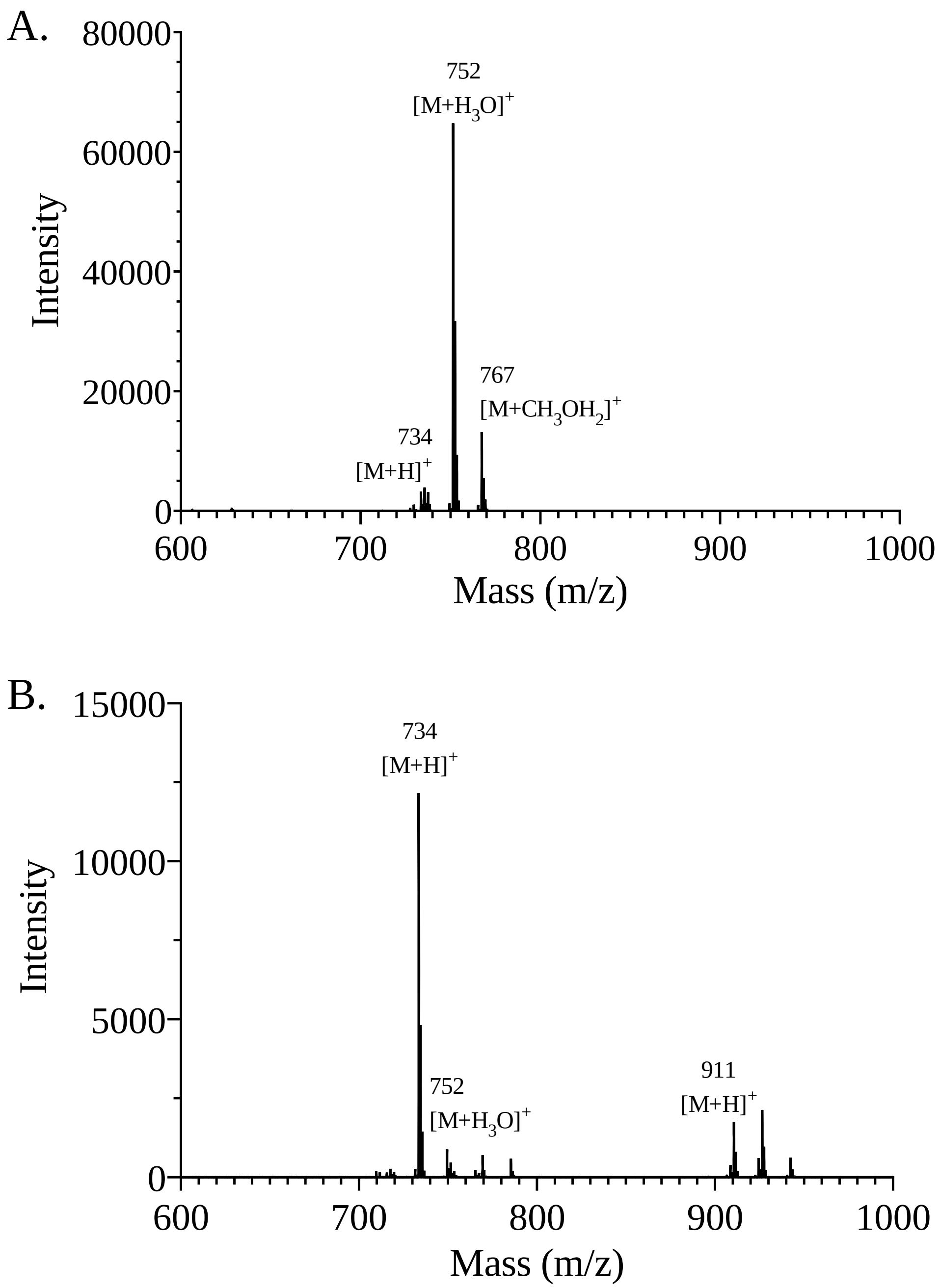


**Figure S2.** MALDI spectra of (**A**) EM and (**B**) AD-EM.

© 2017 by the authors; licensee MDPI, Basel, Switzerland. This article is an open access article distributed under the terms and conditions of the Creative Commons by Attribution (CC-BY) license (http://creativecommons.org/licenses/by/4.0/).
